# Potential Environmental Factors Affecting Oil-Degrading Bacterial Populations in Deep and Surface Waters of the Northern Gulf of Mexico

**DOI:** 10.3389/fmicb.2016.02131

**Published:** 2017-01-10

**Authors:** Jiqing Liu, Hernando P. Bacosa, Zhanfei Liu

**Affiliations:** Marine Science Institute, The University of Texas at Austin, Port AransasTX, USA

**Keywords:** Deepwater Horizon oil spill, Gulf of Mexico, light Louisiana sweet crude oil, petroleum hydrocarbons, oil-degrading bacteria, nutrients, temperature, bacterial community

## Abstract

Understanding bacterial community dynamics as a result of an oil spill is important for predicting the fate of oil released to the environment and developing bioremediation strategies in the Gulf of Mexico. In this study, we aimed to elucidate the roles of temperature, water chemistry (nutrients), and initial bacterial community in selecting oil degraders through a series of incubation experiments. Surface (2 m) and bottom (1537 m) waters, collected near the Deepwater Horizon site, were amended with 200 ppm light Louisiana sweet crude oil and bacterial inoculums from surface or bottom water, and incubated at 4 or 24°C for 50 days. Bacterial community and residual oil were analyzed by pyrosequencing and gas chromatography-mass spectrometry (GC-MS), respectively. The results showed that temperature played a key role in selecting oil-degrading bacteria. Incubation at 4°C favored the development of *Cycloclasticus, Pseudoalteromonas*, *Sulfitobacter*, and *Reinekea*, while 24°C incubations enhanced *Oleibacter, Thalassobius, Phaeobacter, and Roseobacter.* Water chemistry and the initial community also had potential roles in the development of hydrocarbon-degrading bacterial communities. *Pseudoalteromonas*, *Oleibacter*, and *Winogradskyella* developed well in the nutrient-enriched bottom water, while *Reinekea* and *Thalassobius* were favored by low-nutrient surface water. We revealed that the combination of 4°C, crude oil and bottom inoculum was a key factor for the growth of *Cycloclasticus*, while the combination of surface inoculum and bottom water chemistry was important for the growth of *Pseudoalteromonas*. Moreover, regardless of the source of inoculum, bottom water at 24°C was a favorable condition for *Oleibacter.* Redundancy analysis further showed that temperature and initial community explained 57 and 19% of the variation observed, while oil and water chemistry contributed 14 and 10%, respectively. Overall, this study revealed the relative roles of temperature, water chemistry, and initial bacterial community in selecting oil degraders and regulating their evolution in the northern Gulf of Mexico.

## Introduction

The exploration, production, and transportation of crude oil are expected to increase worldwide, since the crude oil demand is projected to grow 50% by 2025 (Hirsch et al., 2005). As a consequence, the frequency of oil spill is likely to increase in the future and oil pollution remains as threat to marine ecosystems. Although oil components are toxic to many marine organisms (Gerdes et al., 2005), a substantial proportion of crude oil can be degraded by certain indigenous microorganisms such as bacteria and fungi. These microorganisms often play a critical role in the weathering of spilled oil in marine environments (Harayama et al., 2004; Atlas and Hazen, 2011). However, biodegradation rates of petroleum hydrocarbons and the development of oil degraders depend on the oil composition and environmental conditions (Leahy and Colwell, 1990; Dubinsky et al., 2013).

The Deepwater Horizon (DWH) oil spill that occurred in April 2010 released 210 million gallons of light sweet crude oil into the northern Gulf of Mexico (nGoM) (Crone and Tolstoy, 2010). This spill was across large environmental gradients when the oil ascended from the deep sea to the upper surface waters, with temperature ranging from about 4–5°C to >20°C, along with variations of pressure, nutrients, solar irradiance, and ambient bacterial communities (Liu et al., 2012; Liu and Liu, 2013). The dramatic changes of environmental conditions in the water column during the vertical rising of oil may be crucial to the succession of indigenous bacteria and the biodegradation processes (Fuhrman et al., 2006; Treusch et al., 2009). In the deep water oil plume with high proportions of low-molecular-weight hydrocarbons and under cold temperature, bacterial communities were dominated by *Oceanospirillales*, *Colwellia*, and *Cycloclasticus* of γ*-Proteobacteria* within 1 month of the spill (Camilli et al., 2010; Hazen et al., 2010; Edwards et al., 2011; Mason et al., 2012; Redmond and Valentine, 2012). However, once oil ascended to sea surface, the bacterial community in the oil slick quickly shifted to different groups of γ*-Proteobacteria* (*Pseudomonas*, *Vibrio*, *Acinetobacter*, *and Alteromonas*), and further to α*-Proteobacteria* on the way to coastal lines (Redmond and Valentine, 2012; Liu and Liu, 2013). Temperature fluctuation from deep water (4°C) to sea surface (∼26°C) and composition variations of natural gas and hydrocarbons from crude oil were attributed as the leading factors to this rapid community shift (Redmond and Valentine, 2012; Dubinsky et al., 2013). This is not surprising since temperature not only affects the metabolic rates of microbes including microbial growth and survival, but also selects the types of bacteria that can adapt to certain temperature ranges. The effect of temperature on crude oil degradation rates and bacterial types has often been observed (Nedwell, 1999; Eriksson et al., 2001; Coulon et al., 2007). For example, two bacterial isolates from Antarctic seawater showed lower hydrocarbon degradation rates at 4°C than at 20°C (Michaud et al., 2004). However, some microbes such as bacteria and fungi from several cold environments have great potentials for hydrocarbon degradation at ∼0°C (Whyte et al., 1996; Margesin et al., 2003).

In our previous work, we demonstrated the crucial role of sunlight in shaping the oil-degrading communities in the nGoM waters by incubating surface water both under the natural sunlight and in the dark (Bacosa et al., 2015b). Other environmental factors such as nutrients, and initial communites, which vary greatly from deep to surface waters, may have also contributed to the bacterial community shifts. For example, nitrate and phosphate are enriched in deep water, but depleted in surface water. Inorganic nutrients are often a limiting factor during hydrocarbon degradation in natural environments, and may regulate the development of oil-degrading bacterial communities (Edwards et al., 2011; Dubinsky et al., 2013; Elango et al., 2014). Ambient bacterial community may also be important as oil encounters different communities during the transit from deep to surface ocean. In the Gulf of Mexico, marine microorganisms are constantly exposed to petroleum hydrocarbons, since the widely distributed natural seeps provide a continuous source of petroleum hydrocarbons to the water (MacDonald et al., 1996; Kvenvolden and Cooper, 2003; Hu et al., 2009). It is therefore not surprising that initial bacterial community, in particular the oil-degrading microorganisms, may be important in controlling the development of oil-degrading communities.

Despite the numerous studies on oil biodegradation after the DWH oil spill, the respective role of these environmental factors on the development of bacterial communities in the presence of oil has not been systematically examined. This knowledge is crucial in predicting fates of oil after oil spills and developing bioremediation strategies. In our recent work, we have demonstrated that solar radiation plays a key role in controlling the fate of the spilled oil and shaping the community structures of oil degraders (Bacosa et al., 2015a,b). The goal of this study is to examine the roles of temperature, nutrients, and initial bacterial community, in the biodegradation of oil and development of oil-degrading bacterial communities. A series of on-deck incubation experiments were set up during the May 2013 research cruise to single out each environmental factor and its contribution to the development of oil degraders in the nGoM waters.

## Materials and Methods

### Seawater Collection and Source Oil

Seawater was collected during the *R/V* Pelican cruise in May 2013 using Niskin bottles mounted on a conductivity-temperature-depth (CTD) rosette (Seabird 911) at a station (28° 44.20′N; 88° 21.82′W) 1.4 km from the DWH site. Water from two depths (2 and 1537 m) was used for the incubation experiments. The temperature, salinity, and dissolved oxygen of surface and bottom waters were 25.1 and 4.4°C, 34 and 35 ppt, and 6.6 and 7.0 mg L^-1^, respectively. The hydrocarbons in the water samples were below detection level (Liu et al., 2014). Light Louisiana sweet crude oil (LLS) was obtained from BP (BP Exploration and Production Inc.) as a surrogate for the Macondo (MC252) crude oil released during the DWH oil spill. The hydrocarbon composition of LLS can be found in our previous work (Bacosa et al., 2015a).

### Incubation Experiments

Incubation experiments were initiated while onboard *R/V* Pelican in May 2013 and continued at the Marine Science Institute of the University of Texas. Approximately 10 L each of surface (2 m) and bottom (1537 m deep) water was collected. Six liters were filtered immediately through 0.2 μm nylon filter (Millipore) to remove the bacteria. Aliquots of filtered water were frozen at -20°C for nutrient analysis. Details of the incubation experiment are described in **Table 1**. Briefly, 20 mL of filtered surface water (SW) or filtered bottom water (BW) was transferred into a series of 30 mL pre-combusted amber bottles using a sterilized graduated cylinder, and then the bottles were inoculated with either 2.5 mL unfiltered bottom water (BI) or 2.5 mL unfiltered surface water (SI) as inoculums. LLS oil was added to each bottle using a microliter syringe made of glass and stainless steel, at a final concentration of ca. 200 mg L^-1^. Parallel incubations without oil addition for each treatment combination were set up as controls. Samples were incubated in the dark at 4°C or at 24°C (similar to the *in situ* water temperature). All vials were manually shaken lightly for about 10 s every other day. The shaking, together with the headspace (∼8 mL) of incubation bottles, ensured that the degradation was under oxic condition.

**Table 1:** Description of the incubation experiments.

	Treatment	Description
(1)	SW+BI_4	SW inoculated with BI (Control), 4°C
(2)	SW+BI_24	SW inoculated with BI (Control), 24°C
(3)	SW+SI+oil_4	SW inoculated with SI+LLS oil, 4°C
(4)	SW+SI+oil_24	SW inoculated with SI+LLS oil, 24°C
(5)	SW+BI+oil_4	SW inoculated with BI+LLS oil at 4°C
(6)	SW+BI+oil_24	SW inoculated with BI+LLS oil, 24°C
(7)	BW+SI _4	BW inoculated with SI (Control), 4°C
(8)	BW+SI _24	BW inoculated with SI (Control), 24°C
(9)	BW+SI+oil_4	BW inoculated with SI+LLS oil, 4°C
(10)	BW+SI+oil_24	BW inoculated with SI+LLS oil, 24°C
(11)	BW+BI+oil_4	BW inoculated with BI+LLS oil, 4°C
(12)	BW+BI+oil_24	BW inoculated with BI+LLS oil, 24°C


*Light Louisiana sweet crude oil (LLS) was directly added at a final concentration of 200 ppm. SW- filtered surface water; SI- unfiltered surface water (inoculum); BW-filtered bottom water; BI- unfiltered bottom water (inoculum). 20 ml of filtered water and 2.5 mL of unfiltered water/inoculum were added. Water samples were filtered through 0.20 μm polycarbonate filters. Incubation was conducted at 4 or 24°C in the dark.*

Five bottles were sacrificed at each time point (0, 5, 12, 30, and 50 days) for each treatment. Three bottles were immediately stored in a freezer (-20°C) until hydrocarbon analysis. The other two bottles were used for the analysis of nutrients, bacterial densities and community structures. One mL of aliquot from each vial was preserved with formaldehyde (3%) and stored at 4°C for bacterial density analysis, and the remaining solution was filtered through a 0.2 μm Nylon filter (25 mm, Osmonics). The filters were stored frozen (-20°C) for bacterial community structure analysis, and the filtrates were frozen for nutrient analyses, including ammonium, nitrite and nitrate, and phosphate.

### Hydrocarbon Extraction and Analysis

The frozen incubation bottles were thawed, extracted, and fractioned according to the established protocol (Wang et al., 2004; Liu et al., 2012). Briefly, the samples were spiked with deuterated hexadecane-*d*_34_ and phenanthrene-*d*_10_, and extracted three times successively with 15 mL of dichloromethane (DCM). The extracts were combined and eluted through a chromatographic glass column packed with 15 g anhydrous sodium sulfate to remove water. The DCM extracts were concentrated by a Rotovap and exchanged with hexane to a final volume of 1 mL for clean-up and fractionation. The 1 mL concentrated extracts were transferred into silica gel chromatographic columns which were dry-packed with 3 g of activated silica gel and topped with 1 cm of anhydrous granular sodium sulfate. The columns were conditioned with 20 ml of hexane, and the concentrated extracts were loaded into the columns. The saturated and aromatic hydrocarbons were fractionated with 12 mL of hexane and 15 mL of benzene in hexane (50% v/v), respectively. The total GC-detectable saturated *n*-alkanes were analyzed with the hexane fraction and the 16 EPA priority polycyclic aromatic hydrocarbons (PAHs) with the benzene in the hexane fraction. Analysis for *n*-alkanes (C_9_–C_34_), pristane (Pr) and phytane (Ph) was performed on a GC with flame ionization detection (Shimadzu GC 2014) with a JW scientific DB5 column (30 m × 0.25 mm; 0.25 μm film thickness). The 16 PAHs were analyzed with GC coupled with mass spectrometry (GC-MS) (Shimadzu QP2010 plus) with a Restek DB5 column (20 m × 0.18 mm; 0.18 μm film thickness). The detailed temperature programs of the columns were described elsewhere (Liu et al., 2012). Quantification for *n*-alkane analysis was based on the internal standard hexadecane-*d*_34_ and external standards of *n*-alkanes. For the analysis of target PAHs, the selected characteristic ions were used for identification and quantitation (Liu et al., 2012). The recovery rates for hexadecane-*d*_34_ and phenanthrene-*d*_10_ were 80.9 ± 13.5 and 90.6 ± 13.1%, respectively.

### Bacterial Enumeration and Nutrient Analysis

The total bacterial cells of preserved samples were counted using an Accuri C6 Flow Cytometer (FCM) system with a laser emitting at 480 mm using the SYBR^®^ Green I nucleic acid stain technique as previously described (Liu et al., 2013). Briefly, samples were stained and detected from their signatures in a plot of 90° side light scatter (SSC-H) vs. green fluorescence (FL1-H). The counting error for duplicate samples was estimated within 35%.

Nitrate and nitrite were measured using the cadmium reduction method, phosphate using ascorbic acid method, and ammonium using indophenol method through a UV–Vis spectrophotometer (Evolution 160, Thermo Scientific) following established procedures (Bolleter et al., 1961; Strickland and Parsons, 1968; Jones, 1984).

### DNA Extraction and Pyrosequencing

DNA extraction and pyrosequencing from the membrane filters were conducted in the Research and Testing Laboratory, Lubbock, TX, USA, following the established procedures (Smith et al., 2010; Liu and Liu, 2013). The DNA extracts were eluted to 30 μL with molecular biology grade water and quantified using a Nanodrop spectrophotometer (Nyxor Biotech, Paris, France). The DNA samples were used for bacterial community analysis via the bacterial tag-encoded FLX amplicon pyrosequencing (bTEFAP). The Eubacterial primers 28F 5′-TTTGATCNTGGCTCAG-3′ and 519R 5′- GTNTTACNGCGGCKGCTG-3′ were used to amplify about ∼500 bp region of 16S rRNA gene (Dowd et al., 2008; Smith et al., 2010). Tag-encoded FLX amplicon pyrosequencing analyses utilized a Roche 454 FLX instrument with Titanium reagents according to the RTL protocols^1^ for bacterial diversity (Smith et al., 2010).

The pyrosequencing raw data were converted into FASTA files, and analyzed using a custom scripted bioinformatics pipeline (Handl et al., 2011; Ishak et al., 2011), including four major steps: quality trimming, clustering, chimera checking, and denoising. Reads over 250 bp are trimmed to Q25 average for the entire read. Trimmed reads were clustered at 4% using USEARCH and any not joining a cluster were removed. Cluster centroids were run through *de novo* chimera checking using UCHIME and all clusters with chimeric centroids were removed. Remaining sequences were globally aligned to their cluster centroid to determine per-base correction based on the alignment and quality of the centroid and query read (Edgar, 2010; Edgar et al., 2011). To determine the identity of each remaining sequence, the sequences were clustered into operational taxonomic unit (OTU) clusters. For each cluster, the seed sequence was put into a FASTA formatted sequence file. This file was then queried against a database originating from NCBI^2^ and gene sequences were identified using Krakenblast^3^. Based upon BLASTn + sequence identity, each bacterium was identified to its closest relative and taxonomic level from fragments of gene sequences. After best hit processing, genus and higher level taxonomic designations were compiled using a secondary post-processing algorithm and relative percentages of bacterial taxa were determined for each individual sample. The percentage of each organism was then analyzed for each sample based on the relative number of reads. The sequences were classified at the appropriate taxonomic levels based on the following criteria: greater than 97% at species level, 95–97% at genus level; 90–95% at family level, 85–90% at order level, 80–85% at class level, and 77–80% at phyla level.

Pyrosequencing of 16S rRNA gene amplicons from the 38 samples resulted in a total of 209,380 quality-filtered sequences for an average of 5,510 sequences per sample. The representative sequences have been deposited in the GenBank under accession numbers KX982530 to KX982551.

### Phylogenetic Investigation of Communities by Reconstruction of Unobserved States (PICRUSt)

Functional gene prediction was assessed using Phylogenetic Investigation of Communities by Reconstruction of Unobserved States (PICRUSt) (Langille et al., 2013). PICRUSt is a bioinformatics tool that allows for the reconstruction of a metagenome by inference of gene content using 16S ribosomal DNA sequences. For the analysis of 16S rRNA gene amplicon, we used MOTHUR^4^ version 1.16.1 (Schloss et al., 2009). Input files for PICRUST were created in MOTHUR using the ‘make.biom’ command. PICRUSt metabolic predictions were carried out on closed OTUs at the 97% similarity level. Metabolic predictions on copy-number normalized OTUs were made through the Galaxy (Goecks et al., 2010) server located at http://huttenhower.sph.harvard.edu/galaxy/.

### Statistical Analyses

Non-metric multidimensional scaling (NMDS) was used to examine the overall patterns of bacterial community structure using Hellinger-transformed relative abundances of bacterial genera (Bacosa et al., 2015b). NMDS was performed using Bray-Curtis dissimilarity distances in PAST software package, V2.17 (Hammer et al., 2001). Treatments were then compared using one-way analysis of similarity (ANOSIM) to verify the significance of the clustering. Redundancy analysis (RDA) using R package “Vegan” (Oksanen et al., 2008) was applied to estimate the effects of oil, temperature, nutrient, and initial bacterial community and their association with bacterial community structure and abundance during the incubation. Partial RDA (pRDA) was also performed to estimate the variance explained by each variable. The first-order biodegradation rate constant (*k*) for total alkanes and total PAHs was calculated as previously described (Bacosa et al., 2010; Bacosa et al., 2015a), and the significance between the treatments was tested by *t*-test.

## Results

### Hydrocarbon Degradation

**FIGURE 1:**
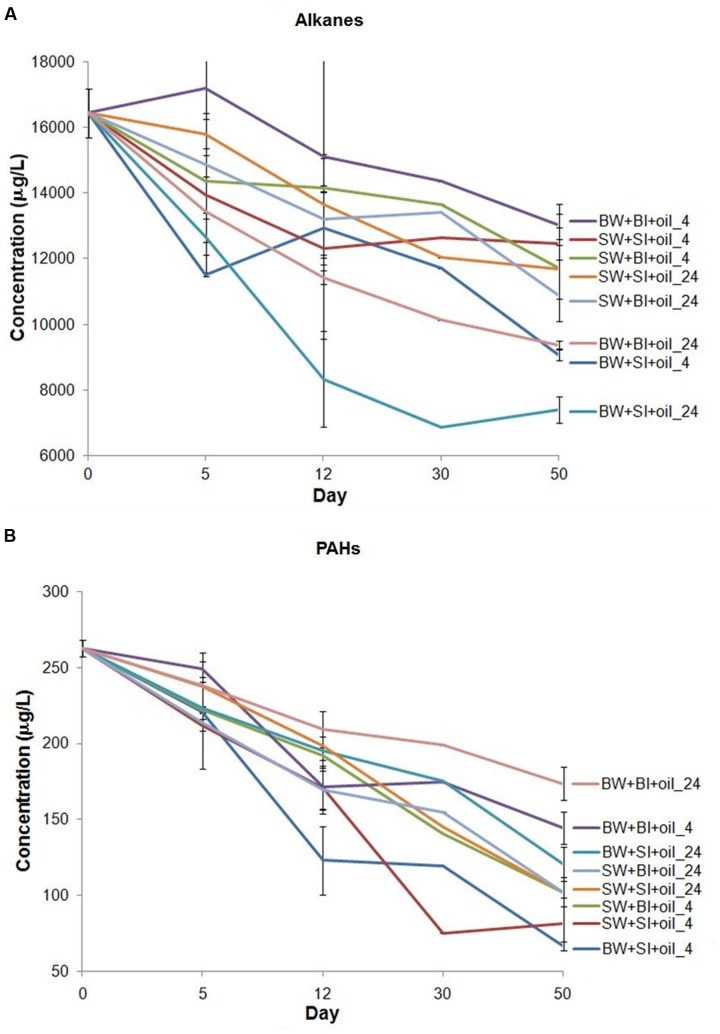
**Changes in the concentration of** **(A)** total *n*-alkanes (C_9_–C_34_), plus pristane and phytane, and **(B)** 16 priority polycyclic aromatic hydrocarbons (PAHs) in the incubation experiments. BW- filtered bottom water, BI- unfiltered bottom water (inoculum), SW- filtered surface water, SI- unfiltered surface water (inoculum). Each treatment was incubated at 4 or 24°C in the dark.

We analyzed *n*-alkanes (C_9_–C_34_) and 16 EPA priority PAHs, two major components of crude oil to represent biodegradation (Liu et al., 2012). Total *n*-alkanes in treatments were generally degraded faster at 24°C (*k* = 0.093 to 0.221 d^-1^) than at 4°C (*k* = 0.064 to 0.117 d^-1^) (**Figure 1A**; **Supplementary Table S1**) (*t*-test, *P* < 0.05). Using bottom water also showed relatively higher alkane degradation rates (*k* = 0.073 to 0.221 d^-1^) than using surface water (*k* = 0.064 to 0.095 d^-1^) (*t*-test, *P* < 0.05). Among all treatments, using bottom water with surface inoculum incubated at 24°C showed consistently the highest alkane degradation rate from 12 to 50 days (*k* = 0.221 d^-1^), while using surface water with bottom or surface inoculum had the lowest degradation rates (*k* = 0.064 to 0.065 d^-1^). However, there is no general pattern on which inoculant yielded higher degradation.

The total PAHs were degraded from 34 to 75% over 50 days of incubation (**Figure 1B**). Interestingly, the degradation pattern of PAHs differed from that of alkanes. For example, concentrations of PAHs decreased faster at 4°C (*k* = 0.155 to 0.339 d^-1^) than 24°C (*k* = 0.101 to 0.235 d^-1^) (*t*-test, *P* < 0.05), the opposite of alkanes. Incubation using surface water also generally produced faster PAHs degradation than that using bottom water. Among all treatments, PAHs degraded the most in the bottom and surface water with surface inoculum incubated at 4°C (*k* = 0.337 to 0.339 d^-1^). Surprisingly, the bottom water with bottom inoculums showed the lowest PAH degradation both at 24°C (*k* = 0.101 d^-1^) and 4°C (*k* = 0.155 d^-1^), even lower than all surface water incubations (*k* = 0.221 to 0.337 d^-1^), indicating that the degradation of PAHs may not be related to the bottom water medium, or water chemistry.

### Bacterial Growth

**FIGURE 2:**
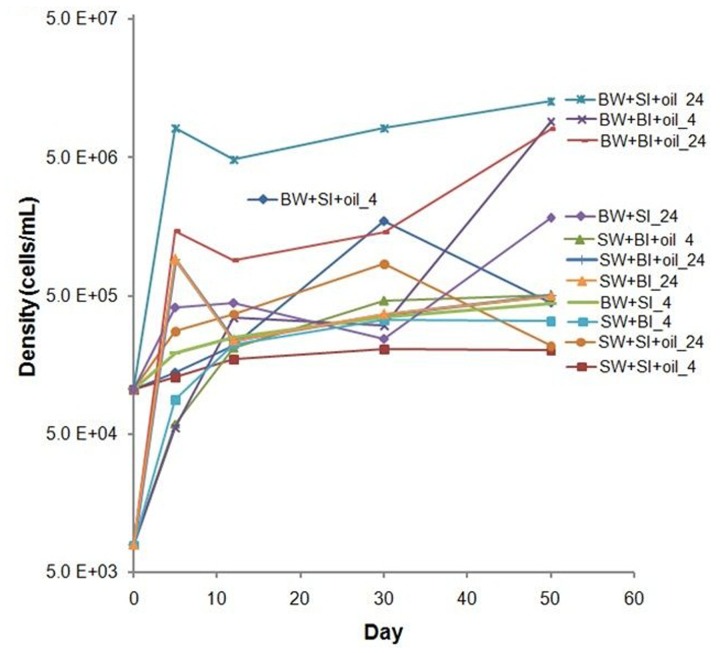
**Changes in bacterial density in incubation experiments.** SW- filtered surface water; SI- unfiltered surface water (inoculum); BW- filtered bottom water; BI- unfiltered bottom water (inoculum). Water samples were filtered through 0.20 μm polycarbonate filters. Incubation was conducted at 4 or 24°C in the dark.

The initial bacterial density was 10-fold lower in bottom water than in surface water; bacterial densities generally increased during the incubation in oil treatments and non-oiled controls, but the increase was higher in oil-amended treatments (**Figure 2**). Inoculation with surface bacteria community resulted in 1–78-fold increase while that with bottom bacteria community was from 8 to 1100-fold. The highest growth using surface inoculum occurred in the treatment with bottom water incubated at 24°C (78–120-fold), while using bottom inoculum occurred in bottom water incubated at 24°C (110–1100-fold). Moreover, using bottom water produced relatively higher cell density. Bacterial density is positively correlated with alkane degradation (*P* = 0.0005) but not with PAH degradation (*P* = 0.98) (**Supplementary Figure S1**).

### Bacterial Community Dynamics

#### Inoculation with Bottom Water Microbial Community (BI)

**FIGURE 3:**
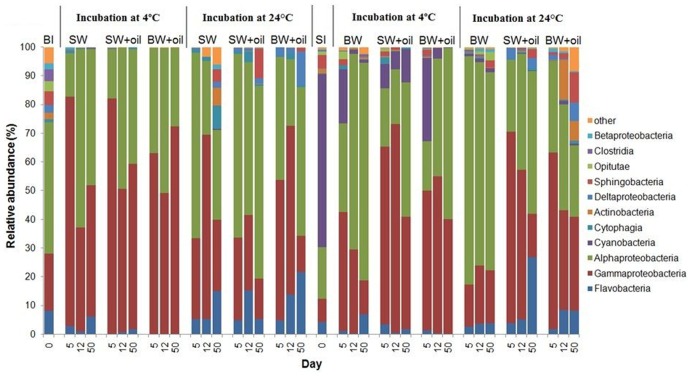
**Changes in the composition of bacteria at class level in incubation experiments with or without crude oil.** Filtered surface water (SW) or filtered bottom water (BW) inoculated with bottom water (1537 m) bacterial community (BI) or with surface water (2 m) bacterial community (SI). Treatments were incubated at 4 or 24°C. Only abundances greater than 2% in at least one of the samples are shown. The numbers on horizontal axis indicate the days of incubation.

The initial bottom water microbial community was primarily composed of α- and γ-*Proteobacteria, Flavobacteria*, *Sphingobacteriia*, *Clostridia*, and *Opitutae* at the class level with relative abundances of 46, 20, 8, 5, 4, and 3%, respectively (**Figure 3**). When incubated at 4°C, the community shifted to mainly α-*Proteobacteria* (38 ± 15%) and γ-*Proteobacteria* (60 ± 16%). In contrast, higher percentages of α-*Proteobacteria* (47 ± 17%) and lower percentages of γ-*Proteobacteria* (34 ± 19%) were found in the 24°C incubations, suggesting that different bacterial phylotypes developed at different temperatures. Several other groups, including *Flavobacteria*, *Deltaproteobacteria, Cytophagia*, and *Sphingobacteria*, also exhibited higher abundances (7–35%) at 24°C.

**FIGURE 4:**
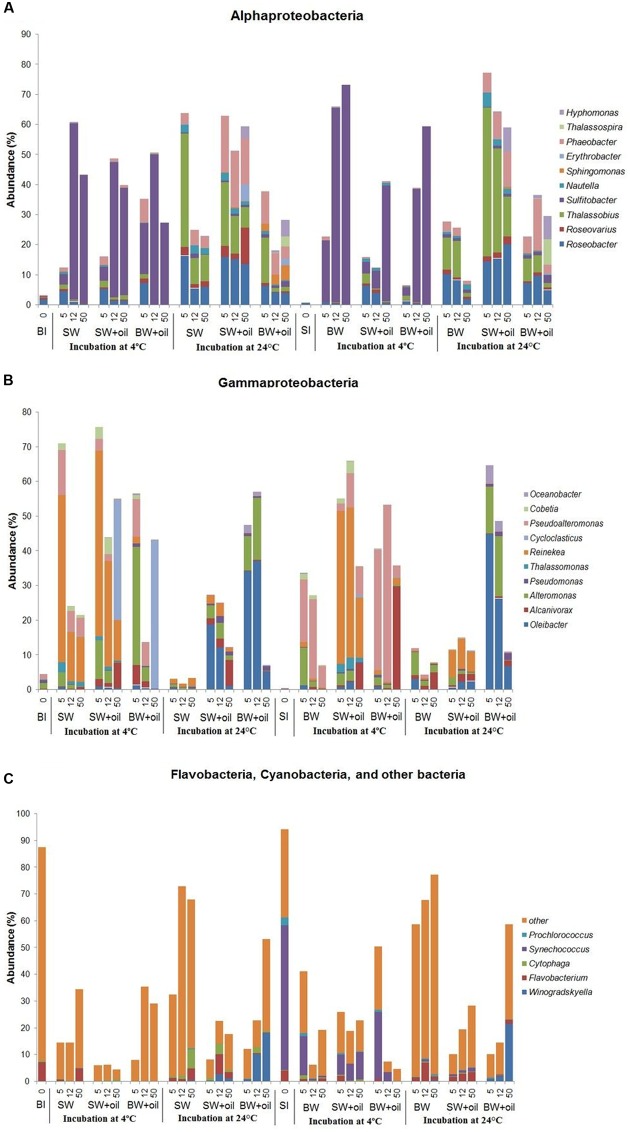
**Changes in the relative abundances of bacteria at genus level in incubation experiments with or without crude oil.** Only abundances greater than 2% in at least one of the samples are shown except for other bacteria. **(A)** *Alphaproteobacteria*, **(B)** *Gammaproteobacteria*, **(C)** *Flavobacteia*, *Cyanobacteria*, and other bacteria. Filtered SW or filtered BW inoculated with bottom bacterial community (BI) or with surface bacterial community (SI). Treatments were incubated at 4 or 24°C. The numbers on horizontal axis indicate the days of incubation.

The striking differences among treatments can be observed at the genus level (**Figure 4**). *Sulfitobacter* predominated the less diverse community of α-*Proteobacteria* at 4°C, while *Roseobacter, Thalassobius, Phaeobacter*, and *Hyphomonas* were abundant in the more diverse communities at 24°C treatments (**Figure 4A**), suggesting the key role of temperature in shaping these groups of bacteria. Interesting patterns were also observed among the members of γ-*Proteobacteria. Cycloclasticus*, a known PAH degrader (Dyksterhouse et al., 1995; Geiselbrecht et al., 1998), dominated on day 50 in 4°C incubations (35–43%), but nearly undetectable in controls and in 24°C treatments (**Figure 4B**). *Alcanivorax*, a known alkane degrader (Bacosa et al., 2015b, 2016), developed from undetectable to 5–7% with oil addition at both temperatures, but not in controls. In the presence of oil, *Alteromonas* increased from 1% initially to 4–11% on days 5 and 12 in surface water, but particularly dominated using bottom water (15–34%), suggesting the importance of water chemistry in controlling their development. Notably, the nearly undetected *Oleibacter* dominated the oiled communities incubated at 24°C (12–34%). *Winogradskyella* also popped out in bottom water with oil.

#### Inoculation with Surface Water Microbial Community (SI)

The initial bacterial community from 2 m surface water (SI) was dominated by *Cyanobacteria* (60.4%), which was greatly reduced (10–30%) in 4°C oiled treatments but still abundant throughout the end of incubation (**Figure 3**). However, these *Cyanobacteria*, mainly *Synechococcus* (**Figure 4C**), were nearly undetected in 24°C treatments from 5 days. In contrast to BI, SI-inoculated treatments did not show any evident differences between the patterns of α*-Proteobacteria* and γ-*Proteobacteria* in 4 and 24°C. At 4°C with oil additions, the dominant group shifted from γ-*Proteobacteria* (at 5 and 12 days) to α-*Proteobacteria* at 50 days, while α-*Proteobacteria* dominated the control throughout the incubation. A similar pattern was observed in 24°C treatments, except for the presence of *Flavobacteria, Actinobacteria*, and *Sphingobacteria.*

Bacterial communities in both control and oiled treatments were distinct from the initial seawater communities. Also, those incubated at 4 and 24°C treatments differed greatly from each other (**Figure 4**). Among the α-*Proteobacteria*, *Sulfitobacter* was the most dominant genus in 4°C incubations which increased up to 120-fold, while incubations at 24°C were represented by *Roseobacter, Thalassobius*, and *Phaeobacter*, similar to what was observed using bottom inoculums (**Figure 4A**). At 4°C, *Alcanivorax* reached 30% in the bottom water at 50 days, and 8% in the surface water, compared to <1% in the control. *Pseudoalteromonas*, another commonly known oil degrading bacteria (Brakstad and Lødeng, 2005; Bacosa et al., 2016) grew rapidly from <1 to 35% on 5 days, and 51% on 12 days in the oil treatments (**Figure 4B**). Their growth was also observed in controls but to a lesser degree (6–23%). *Oleibacter* and *Alteromonas* predominated in 24°C incubation with oil while *Reinekea* was observed in both temperatures.

### Statistical Analysis

**FIGURE 5:**
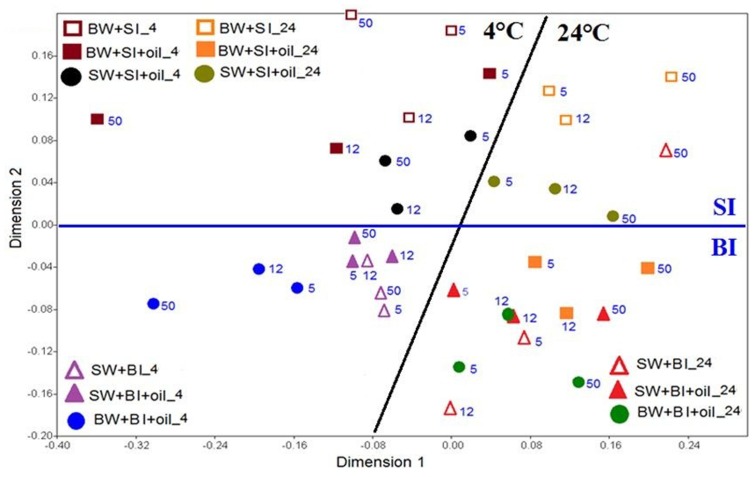
**Non-metric multi-dimensional scaling (NMDS) analysis of bacterial communities based on Bray-Curtis coefficient matrix of Hellinger-transformed relative abundances of bacterial genera incubated at 4 and 24°C.** The numbers indicated the days of incubation. The significance of clustering was analyzed by one-way analysis of similarities (ANOSIM). BW, filtered bottom water; BI, unfiltered bottom water (inoculum); SW, filtered surface water; SI, unfiltered surface water (inoculum).

The similarity of the microbial communities was examined with non-metric multidimensional scaling (NMDS), and the significance among the treatments was further tested with analysis of similarity (ANOSIM). The NMDS plot showed that the bacterial community structures using bottom inoculum (BI) (both with oil and control) clustered into two groups, which were mainly driven by temperature (**Figure 5**). In the 4°C experiments, the surface water incubation with added oil clustered the closest with the controls, which separated slightly from the bottom water incubation (with oil addition), especially at day 50. The bacterial community structures in the 24°C experiments clustered closely for all treatments and controls, except for the bottom water incubation control at day 50. A very similar pattern was observed for the treatments with surface inoculums. The temperature again was the main factor of affecting the bacterial community structures. Taken together, the microbial communities developed at 4°C varied significantly from that of 24°C (ANOSIM, *P* = 0.0001) (**Supplementary Figure S2A**). Using surface water and bottom water also produced significantly different communities (ANOSIM, *P* = 0.0141) (**Supplementary Figure S2B**). Moreover, the communities developed from initial bottom inoculum and surface inoculum also varied significantly (ANOSIM, *P* = 0.0001) (**Supplementary Figure S2C**).

**FIGURE 6:**
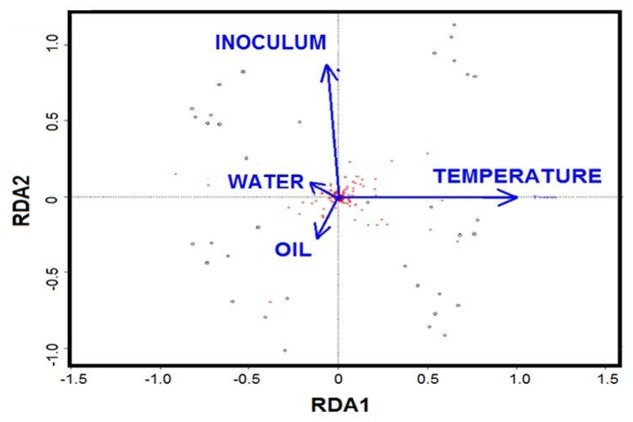
**Biplot diagram of the redundancy analysis (RDA) on microbial community constrained by the presence of oil, temperature, nutrients and initial community**.

Redundancy analysis was applied to evaluate the role of individual factors for all the treatments. The RDA biplot showed that temperature and initial community are the key drivers, while oil and nutrients played minor roles (**Figure 6**). Partial RDA further revealed that temperature was the major driver in shaping the bacterial communities (57%, *P* = 0.001), followed by the initial community with 19% (*P* = 0.007) of the total variation (**Supplementary Table S2**). The additions of oil contributed 14% (*P* = 0.016) of the variation, while water chemistry only accounted for about 10% of the variation.

## Discussion

### The Changes of Hydrocarbons and Bacterial Densities during the Incubation

Our result showed that the bacterial cell density is correlated with degradation of alkanes, but not of PAHs (**Supplementary Figure S1**). The light Louisiana oil contains 74% saturated hydrocarbons (including *n*-alkanes) and 16% aromatic hydrocarbons (including PAHs) (Reddy et al., 2012). Owing to high proportion of alkanes in crude oil, an observable change in total alkane concentration can be usually attributed to an increase in bacterial density (Bacosa et al., 2015b). Since the 16 PAHs comprised only a small fraction of the oil (<2% by weight) and PAHs can be inhibitory to some bacteria (Bacosa et al., 2012, 2015a), an increase in bacterial density sometimes does not provide direct evidence of PAH degradation, rather a shift to PAH-degrading community is more informative (Bacosa et al., 2010, 2012). Furthermore, the abundance of PAH-degrading genes is another useful indicator of PAH degradation (Bacosa and Inoue, 2015). PICRUSt analysis predicted some genes associated with the degradation of aromatic compounds and benzoate (**Supplementary Figure S3**), which is a common metabolite of bacterial metabolism of several PAHs (Haritash and Kaushik, 2009). There is also a significant positive correlation of the total PAH degradation and the abundance of both genes involved in PAHs and benzoate degradation (*P* < 0.01) (**Supplementary Figure S4**), which suggests that the general increase in the copy number of these genes led to increased metabolism of PAHs by bacterial communities.

Alkanes generally disappeared much faster at 24°C than at 4°C, while PAHs were degraded faster at 4°C; this pattern agrees with the previous findings using bacterial cultures from the DWH site (Campo et al., 2013). We observed that the degradation of alkanes decreased with increasing chain length for most treatments (data not shown), indicating the recalcitrance of long-chain alkanes (Harayama et al., 2004; Bacosa et al., 2010). The ratio of C_17_/pristane as indicators of biodegradation, decreased with time in most treatments (**Supplementary Figure S5A**). As to individual PAHs, naphthalene, acenaphthylene, acenaphthene, fluorene, and phenanthrene clearly decreased during the incubation, (data not shown). However, the four-ring chrysene was exceptionally resistant to weathering and degradation (Wang et al., 2004; Liu et al., 2012). The ratios of phenanthrene/chrysene clearly decreased during the incubations, indicating the role of biodegradation (**Supplementary Figure S5B**) (Pastor et al., 2001).

In this study, tremendous shifts of bacterial community structure and decrease of diversity were observed in all oiled treatments (**Supplementary Figure S6**). The Shannon-Wiener index in initial BI community was reduced from 6.4 to <3, and SI from 3.8 to <2.8 in all oiled treatments. This could be mainly due to enrichment and selection of oil-degrading bacteria, toxicity of crude oil components, competition to particular hydrocarbon substrates, or a combination of environmental factors (Bacosa et al., 2012; McGenity et al., 2012; Zeglin, 2015).

### The Roles of Potential Environmental Factors

Temperature fluctuation was the main factor leading to bacterial community shifts from the deep sea plume to surface slick during the DWH oil spill (Redmond and Valentine, 2012; Liu and Liu, 2013). Consistently, among the environmental parameters examined here, temperature has the most profound effect on the bacterial communities during oil degradation, and explains 57% of the data variance. Temperatures of 4 and 24°C particularly select psychrophile and mesophile, respectively (Coulon et al., 2005; Kirchman, 2012). Bacterial genera developed in the 4°C incubations included *Cycloclasticus, Pseudoalteromonas*, *Sulfitobacter* and *Reinekea.* In contrast, the dominant bacteria in the 24°C incubations included *Oleibacter, Thalassobius, Phaeobacter*, and *Roseobacter*. Some bacteria developed well in both 4 and 24°C such as *Alcanivorax* and *Alteromonas*. Incubation at 24°C also increased the bacterial diversity particularly that of using bottom inoculum. Even though with very similar amount of oil degraded, it is worthy to note that *Synechococcus* and *Cyanobacteria* in general, thrived well and still abundant in 4°C treatments for the entire incubation period but were nearly undetected when incubated at 24°C after 5 days. This suggests that higher temperature also increases the toxicity of the oil to sensitive bacteria such as *Cyanobacteria*, which agrees with the findings of Gaur and Singh (1991) on *Anabaena*.

*Alcanivorax* is a known alkane degrader (Harayama et al., 2004; Kostka et al., 2011; Bacosa et al., 2015b, 2016), and its highest abundance of 30% at 50 days corresponded to the highest alkane degradation rate among the 4°C treatments in BW+SI+oil_4. Notably, BW+SI+oil_24, and BW+BI+oil_24 gave the highest alkane degradation rates, as well as the highest abundance of *Oleibacter* (37–45%), suggesting the key role that this genus played in the disappearance of alkanes (Gutierrez et al., 2013). *Sulfitobacter* and *Reinekea* are not known in oil degradation and both were enhanced greatly by 4°C incubation with or without oil, suggesting their limited role in oil degradation, even though some evidence indicated the abundance of *Sulfitobacter* increases with the oil additions (Jung et al., 2010). *Cycloclasticus* popped out in BI-inoculated treatments at 4°C suggesting its role for PAH degradation (Dyksterhouse et al., 1995; Geiselbrecht et al., 1998; Kasai et al., 2002a; Gutierrez et al., 2013). We observed that highest degradation of PAHs in BW+SI+oil_4 corresponds to the highest abundance of *Pseudoalteromonas* among the treatments (36–51%), indicating the important of this bacteria in naphthalene and phenanthrene degradation (Kostka et al., 2011; Dubinsky et al., 2013; Gutierrez et al., 2013; Bacosa et al., 2016). It is worthy to note that *Cycloclasticus* and *Pseudoalteromonas* were enriched for nearly 3 months in the subsurface plumes of dispersed oil during DWH spill, which has a temperature of around 4°C. In our previous experiment (Bacosa et al., 2015b), *Pseudoalteromonas* was also detected in a very low abundance but never became dominant in either light or dark when incubated at natural surface temperature (average of ca. 28°C). Thus, these *Cycloclasticus* and *Pseudoalteromonas* seem to be largely stimulated by the presence of oil in cold environment. Moreover, members of *Cycloclasticus* are known obligate aromatic hydrocarbon degraders in marine environments (Dyksterhouse et al., 1995; Geiselbrecht et al., 1998; Kasai et al., 2002a), indicating that the PAHs in crude oil, combined with exposure to low temperature, mainly determine their success in oil-polluted waters. Surprisingly, *Colwellia*, the common known oil-degraders in deep water oil plume during the DWH spill (Hazen et al., 2010; Mason et al., 2012), were detected in low abundance in our experiments. This is mainly attributed to the evaporation of gaseous components in crude oil as a result of long-term storage as *Colwellia* is usually favored by natural gas components of crude oil (Valentine et al., 2010; Redmond and Valentine, 2012).

The initial bacterial community in both surface water and bottom water consisted of diverse genera. The role of initial community as the seed is a major factor in determining what bacteria developed in the oiled treatments and explained 19% of the data. Some bacterial genera known to be involved in oil degradation were either undetected or in very low abundance (<1%) in both initial seawaters, such as *Alcanivorax*, *Alteromonas*, *Cycloclasticus*, *Pseudoalteromonas*, *Oleibacter*, *Thalassobius*, and *Roseobacter*. For example, *Cycloclasticus* appeared normally at low abundance (<0.3%) in the bottom sea water as cold-tolerant organisms. When both filtered surface and bottom waters were inoculated with bottom bacterial community, *Cycloclasticus* increased remarkably when incubated with oil only at 4°C. This observation indicated that a combination of 4°C and bottom community inoculation was a key factor for the growth of *Cycloclasticus*. Moreover, *Oleibacter*, known to degrade alkanes, was undetected in both surface and bottom water but increased to as high as 37–45% in treatments with oil incubated at 24°C using bottom water. These results indicate that the types of oil degraders developed are selected by certain combined environmental conditions. *Roseobacter* and *Alteromonas*, known to be particle-associated bacteria (Dang and Lovell, 2016), may be linked to the colonization of the oil-water interface.

The initial bottom water was enriched with nitrogen (${NO}_{3}^{-}$+${NO}_{2}^{-}$, 10.5 μM) and phosphorus (PO_4_^3-^, 1.4 μM), while the surface water was nutrient-depleted (${NO}_{3}^{-}$+${NO}_{2}^{-}$, 0.03 μM; PO_4_^3-^, 0.29 μM) (**Supplementary Figure S7**). The difference in nutrients, possibly together with other trace elements, explained 10% of the data variance of bacterial communities among the treatments (**Figure 6**). Bacterial communities in non-oiled control also changed remarkably at 5 days especially the bottom water with surface inoculants. The concentration of nitrate+nitrite increased in treatments at 5 days particularly for the surface inoculants, possibly because the inoculation using whole water not only introduced bacteria but also dissolved and particulate organic matter, such as algal cells, from surface water, which were quickly remineralized. In contrast, concentrations of phosphates in bottom water medium all decreased at 5 days and then remained relatively constant (**Supplementary Figure S7**), suggesting that phosphate was taken up by fast-growing bacteria (Liu and Liu, 2016). Edwards et al. (2011) concluded that despite the dearth of dissolved nutrients, microbes have the potential to degrade a large fraction of oil slick, and addition of nutrients promotes rapid microbial activity. Unlike the oil slick with outside and inside components, the 200 ppm concentration we used in this study is more of an oil sheen that is more accessible to hydrocarbon degrading bacteria, leading to more dramatic changes in bacterial community structure.

In a recent paper, Hazen et al. (2016) asserted that nutrient might select for different hydrocarbon-degrading organisms. Interestingly, different bacteria developed between surface and bottom water incubations. *Alteromonas*, *Pseudoalteromonas*, *Oleibacter*, and *Winogradskyella* developed better in the bottom water than surface water (**Figure 4B**), which suggests that high levels of nutrients may play a key role in the development of these bacteria. This is in good agreement with that of Gutierrez et al. (2013) who used a nutrient rich ONR7a medium for DNA-SIP experiment using surface bacterial community near the DWH site, where *Alteromonas* comprised 90% of the clone library with naphthalene, while *Pseudoalteromonas* and *Oleibacter* comprised 3% of that with phenanthrene and hexadecane, respectively. In contrast, *Reinekea* grew well in the surface water inoculated with both surface and bottom bacterial community, incubated at 4°C (**Figure 4B**). *Thalassobius* also developed well in the surface water incubated at 24°C. These results suggest that nutrients may not limit for *Reinekea* and *Thalassobius*, or lower nutrient concentration even favored their growth. Interestingly, *Alcanivorax* were detected in both oiled treatments and non-oiled control at 4 and 24°C, which indicated that the nutrient concentration was not a limiting factor for these bacteria. *Alcanivorax* usually cannot develop and could be easily out-competed by other bacteria under nutrient-depleted conditions (Kasai et al., 2002b; Hara et al., 2003).

The deep water treatments in this study were under atmospheric pressure, but the role of pressure cannot be discounted with regards to the DWH spill, which occurred at the depth of 1500 m with a hydrostatic pressure of 15 MPa. Schedler et al. (2014) reported that the alkane-degrading *Rhodococcus* grew well at this pressure, while the aromatic-degrading *Sphingobium* was highly affected but still able to degrade naphthalene. In a recent report, Scoma et al. (2016) demonstrated that pressure reduced the growth of two species of *Alcanivorax* significantly and concluded that pressure can shape structure of microbial communities after the oil spill. However, these isolates were adapted to atmospheric pressure, and would naturally respond when subjected to increased pressure during incubation experiment. We do not understand yet, how those bacteria inhabiting the deep-sea and sea floor which are highly adapted to such high pressure environment respond to oil spill.

## Conclusion

In this study we showed that temperature profoundly affected the development of bacterial community after oil exposure in the Gulf of Mexico water. Water chemistry mainly nutrients, and initial bacterial communities were also potential factors in selecting petroleum hydrocarbon-degrading bacteria. We showed that 4°C favored the development of *Cycloclasticus, Pseudoalteromonas*, *Sulfitobacter*, and *Reinekea*, while 24°C incubations enhanced *Oleibacter, Thalassobius, Phaeobacter*, and *Roseobacter. Pseudoalteromonas*, *Oleibacter*, and *Winogradskyella* developed better in the nutrient-rich bottom water, while *Reinekea* and *Thalassobius* were favored by nutrient-poor surface water. This study revealed that the combination of temperature, water chemistry, and initial community are among the potential factors affecting the development of oil-degrading communities in oil-polluted surface and bottom waters in the nGoM, and temperature as the most important one. These results also provide insights in the ecology of oil-degrading microorganisms and help guide future bioremediation strategies for the clean-up of oil spills in the GoM. This study also raises intriguing questions about ecological function of different groups of hydrocarbonoclastic bacteria and unknown hydrocarbon degraders.

## Author Contributions

JL: Conceived and performed the experiment, and wrote the paper. HB: Analyzed the data and wrote the paper. ZL: Conceived the experiment and wrote the paper.

## Conflict of Interest Statement

The authors declare that the research was conducted in the absence of any commercial or financial relationships that could be construed as a potential conflict of interest.

## Footnotes

1 http://www.researchandtesting.com

2 http://ncbi.nlm.nih.gov

3 http://www.krakenblast.com

4 https://www.mothur.org/
