# Supplementary material for: Potential Environmental Factors Affecting Oil-Degrading Bacterial Populations in Deep and Surface Waters of the Northern Gulf of Mexico
Source: Front Microbiol. 2017 Jan 10;7:2131. doi: 10.3389/fmicb.2016.02131 (PMC5222892; doi:10.3389/fmicb.2016.02131)
Supplement: Supplementary file 1 [file Presentation_1.PDF]

SUPPLEMENTARY MATERIAL

TABLE S1. First-order biodegradation rate constants for total alkanes and total PAHs.

Total alkanes	k day^{-1}	R^2	Total PAHs	k day^{-1}	R^2
BW+SI+oil_24	0.221	0.86	BW+SI+oil_4	0.339	0.94
BW+BI+oil_24	0.141	0.97	SW+SI+oil_4	0.337	0.89
BW+SI+oil_4	0.117	0.74	SW+BI+oil_4	0.249	0.97
SW+SI+oil_24	0.095	0.96	SW+SI+oil_24	0.235	0.95
SW+BI+oil_24	0.093	0.91	SW+BI+oil_24	0.221	0.96
BW+BI+oil_4	0.073	0.90	BW+SI+oil_24	0.182	0.94
SW+SI+oil_4	0.065	0.72	BW+BI+oil_4	0.155	0.90
SW+BI+oil_4	0.064	0.86	BW+BI+oil_24	0.101	0.99

TABLE S2. Partial redundancy analysis (pRDA) to determine how much of variation in microbial community composition is explained by statistically significant variables.

**Variables**	**Variation explained (%)**	**P-value**
Oil	14	0.016 *
Temperature	57	0.001**
Nutrient	10	0.094
Inoculum	19	0.007**

FIGURE S1. Correlation of bacterial cell density and degradation of (A) total alkanes and (B) total PAHs.

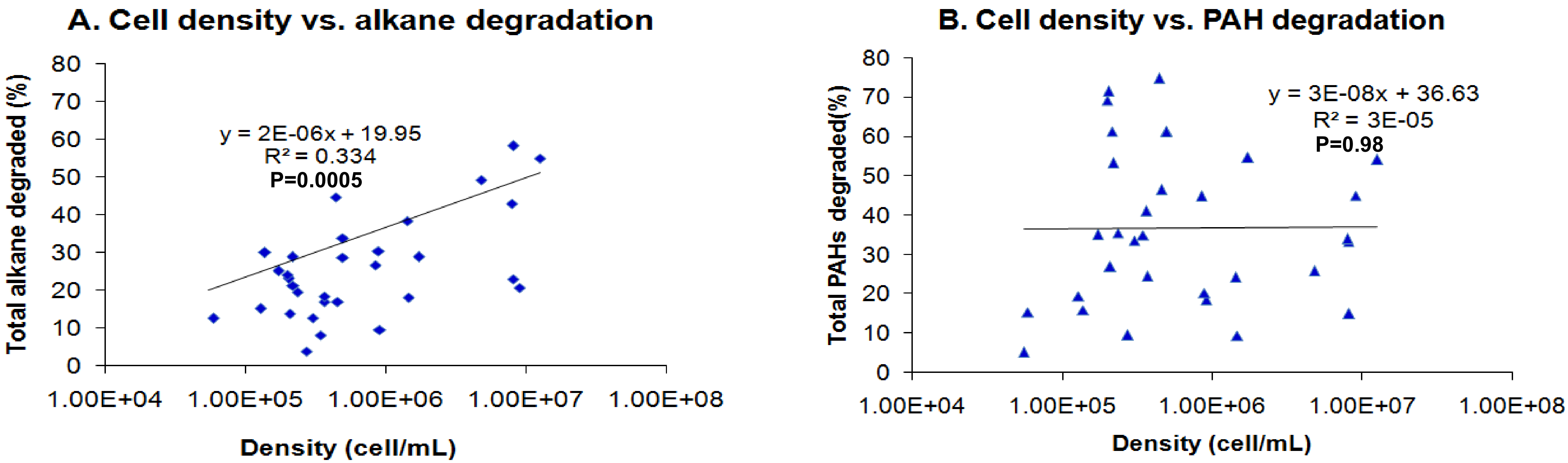

Figure S2. NMDS analysis of bacterial communities and pairwise comparison of bacterial communities based on ANOSIM with the Bray-Curtis distance for (A) temperature (B) nutrients, and (C) initial microbial community or inoculum .

A. Temperature (4ºC vs 24ºC)

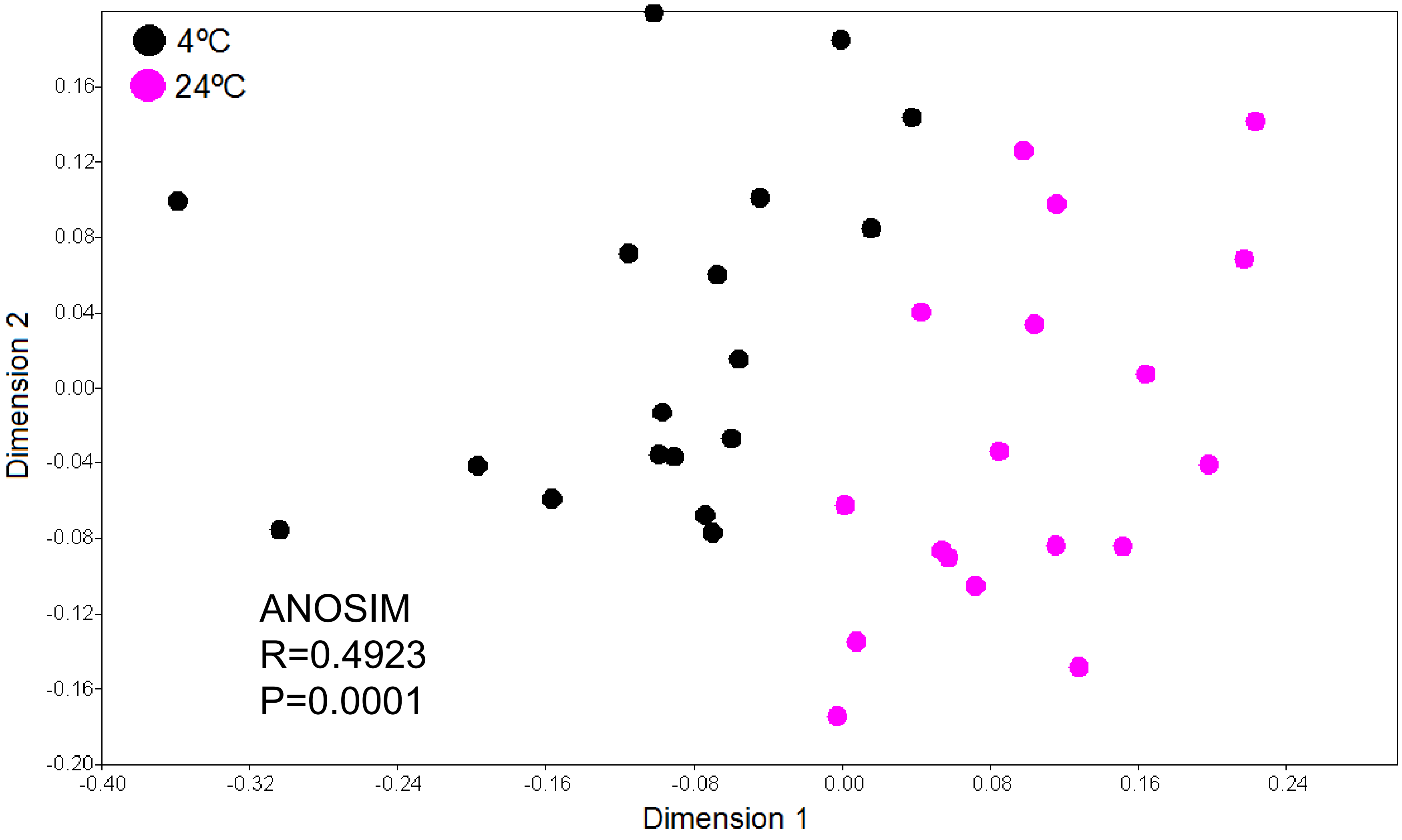

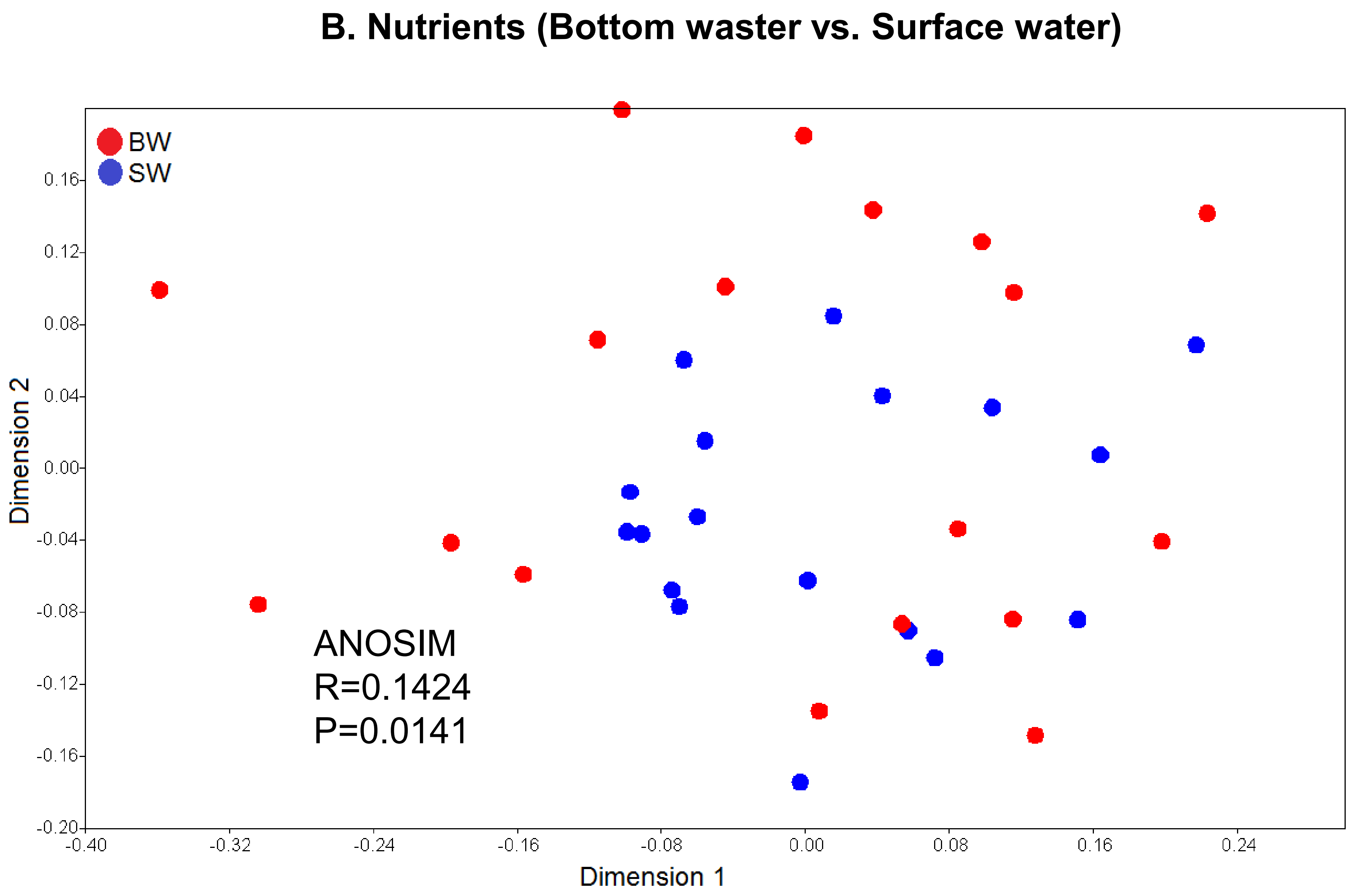
B. Nutrients (Bottom waster vs. Surface water)
BW
SW
ANOSIM
R=0.1424
P=0.0141
Dimension 2
Dimension 1
0.16
0.12
0.08
0.04
0.00
-0.04
-0.08
-0.12
-0.16
-0.20
-0.40
-0.32
-0.24
-0.16
-0.08
0.00
0.08
0.16
0.24

C. Initial microbial community (Bottom inoculum vs. surface inoculum)

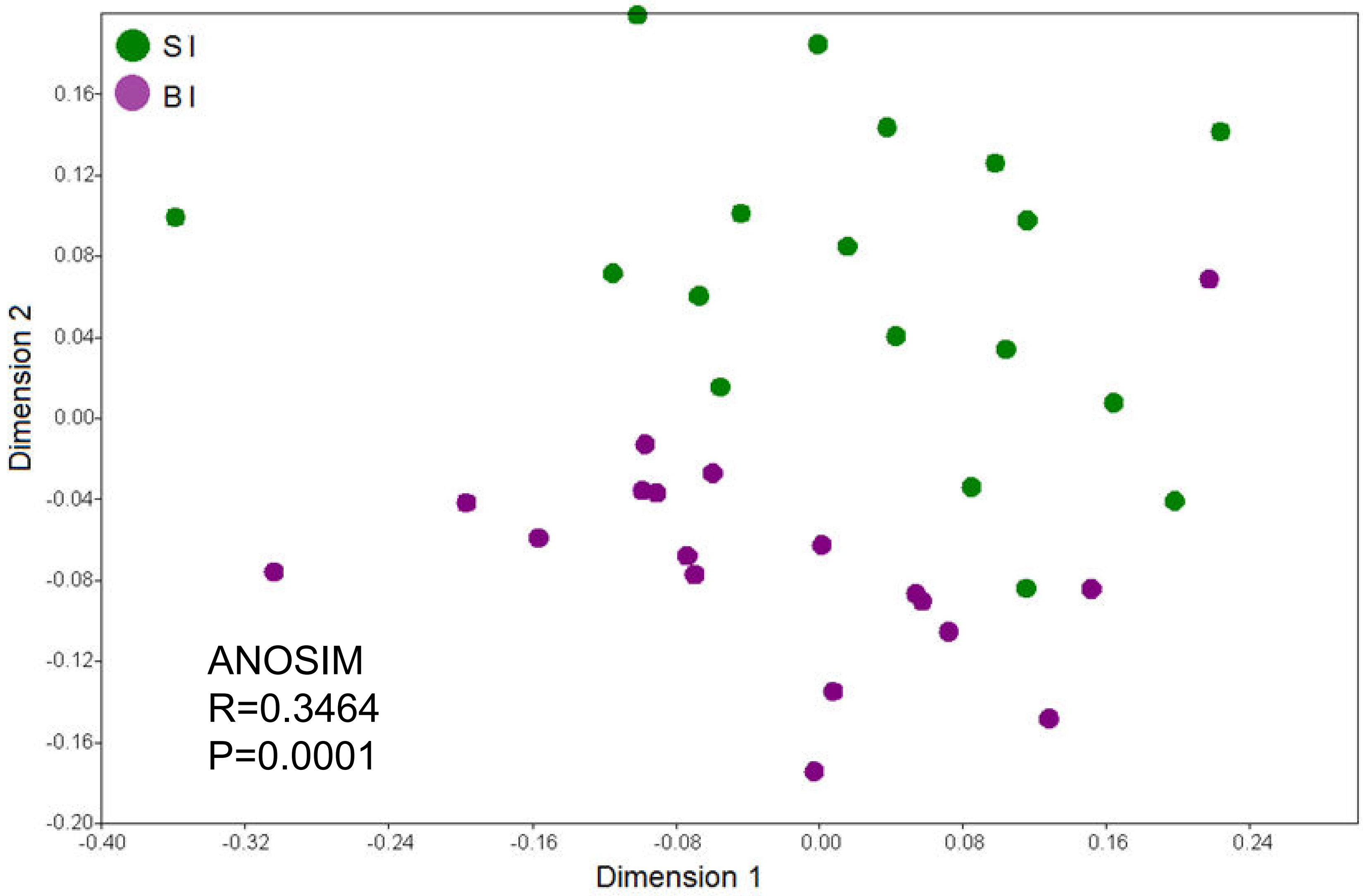

FIGURE S3. Abundance of genes associated with hydrocarbon degradation as predicted by PICRUSt.

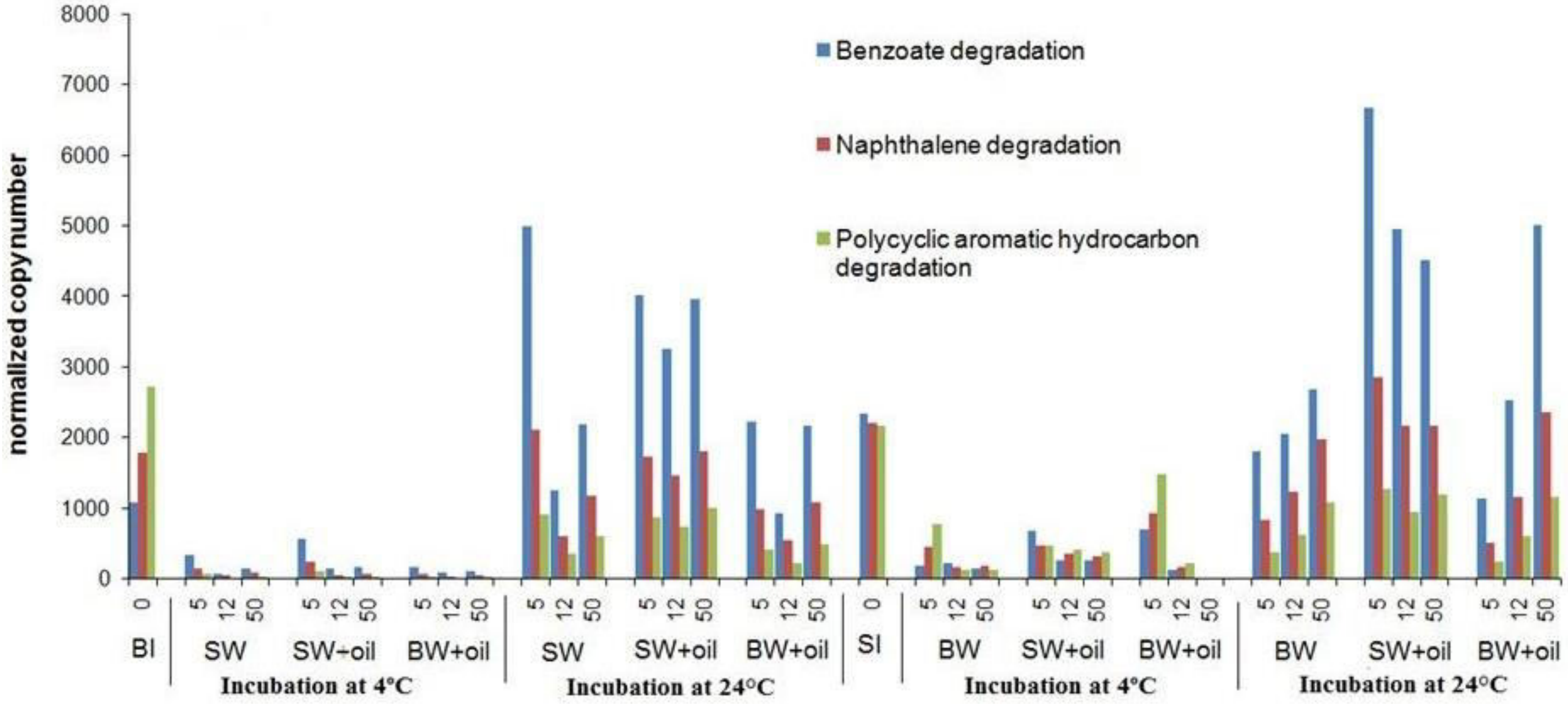

FIGURE S4. Correlation of the abundance of genes related to (A) PAHs degradation, and (B) benzoate degradation plotted against total PAHs degraded.

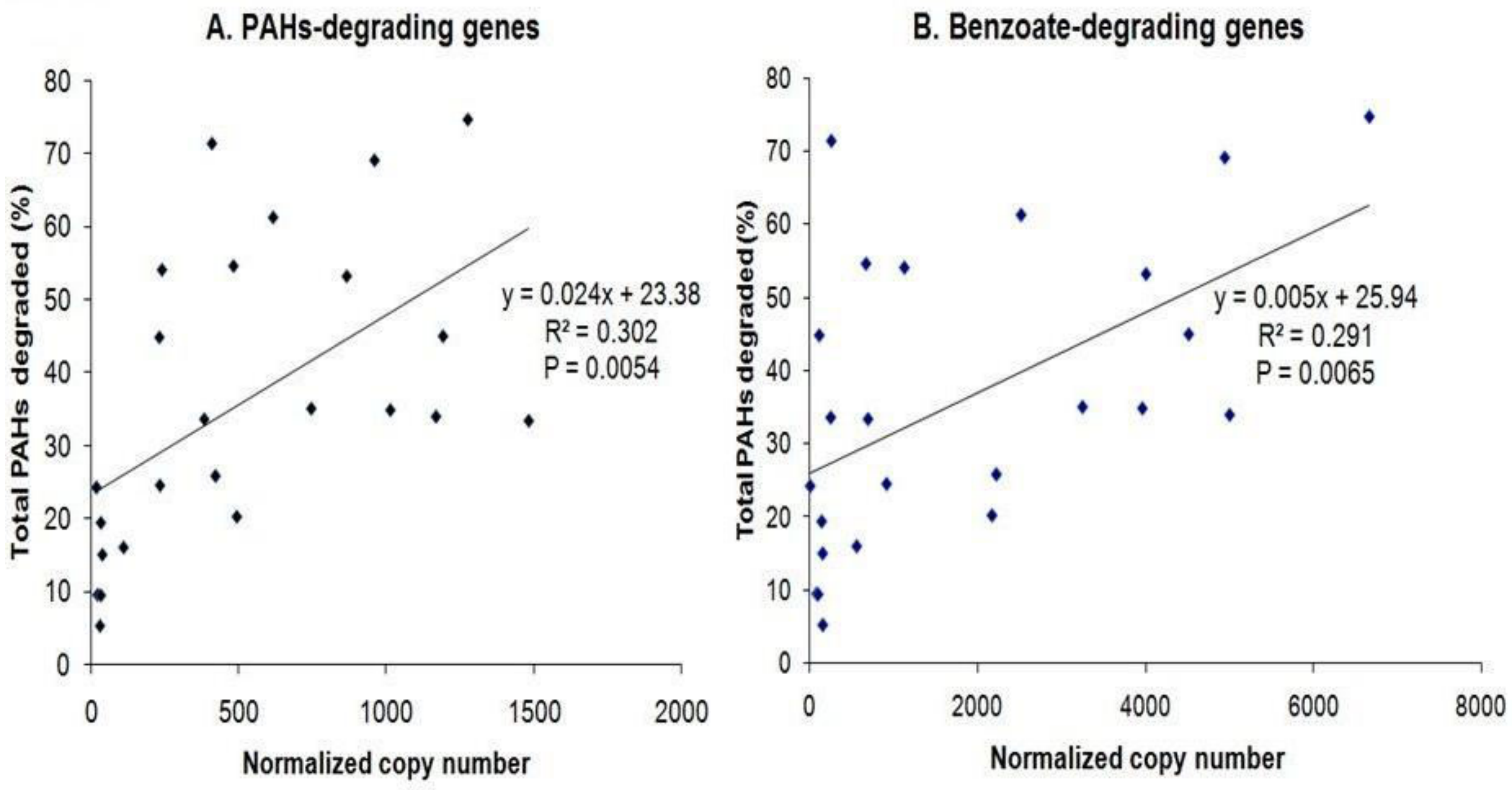

FIGURE S5 . The ratios of (A) C17/pristane and (B) phenanthrene/chrysene at 4°C and 24°C during incubation.

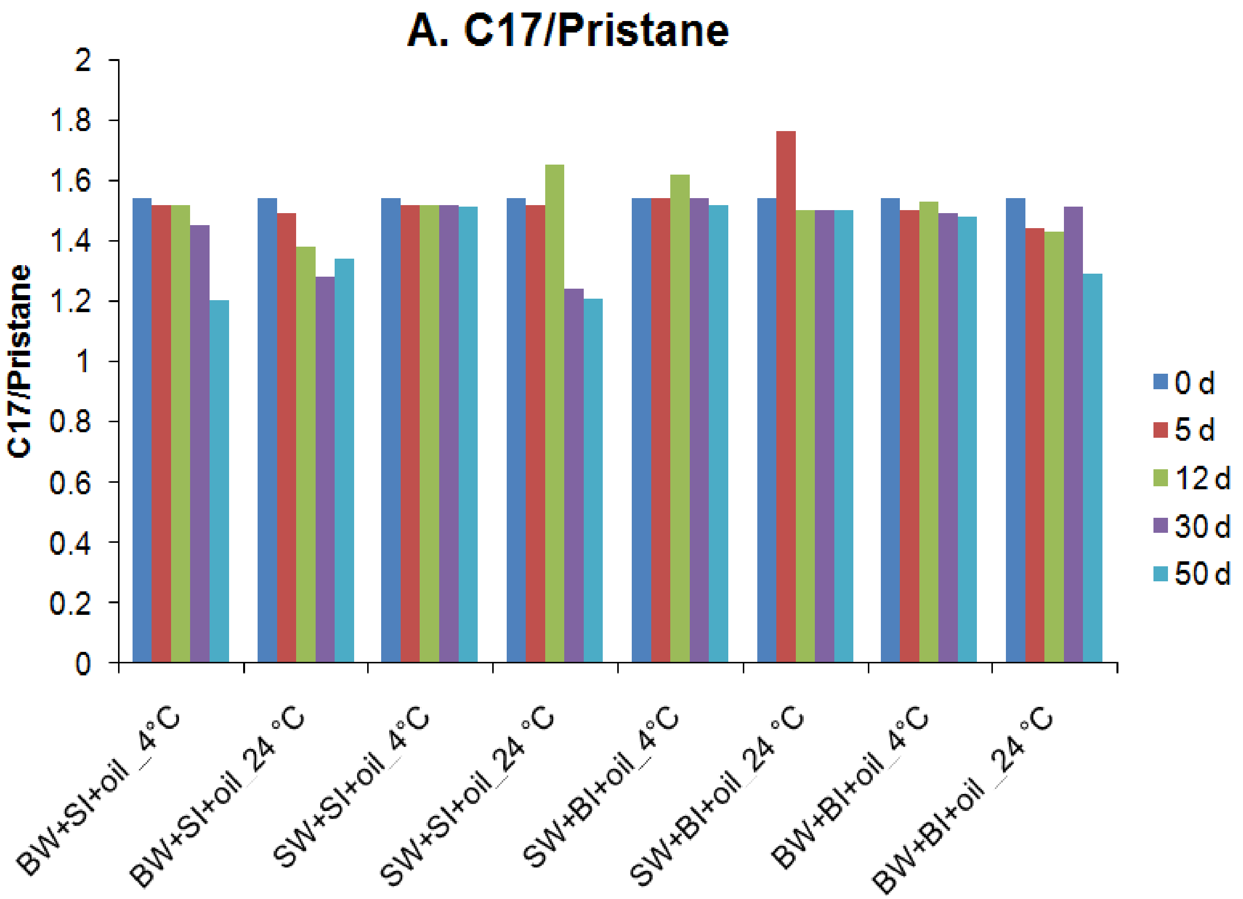

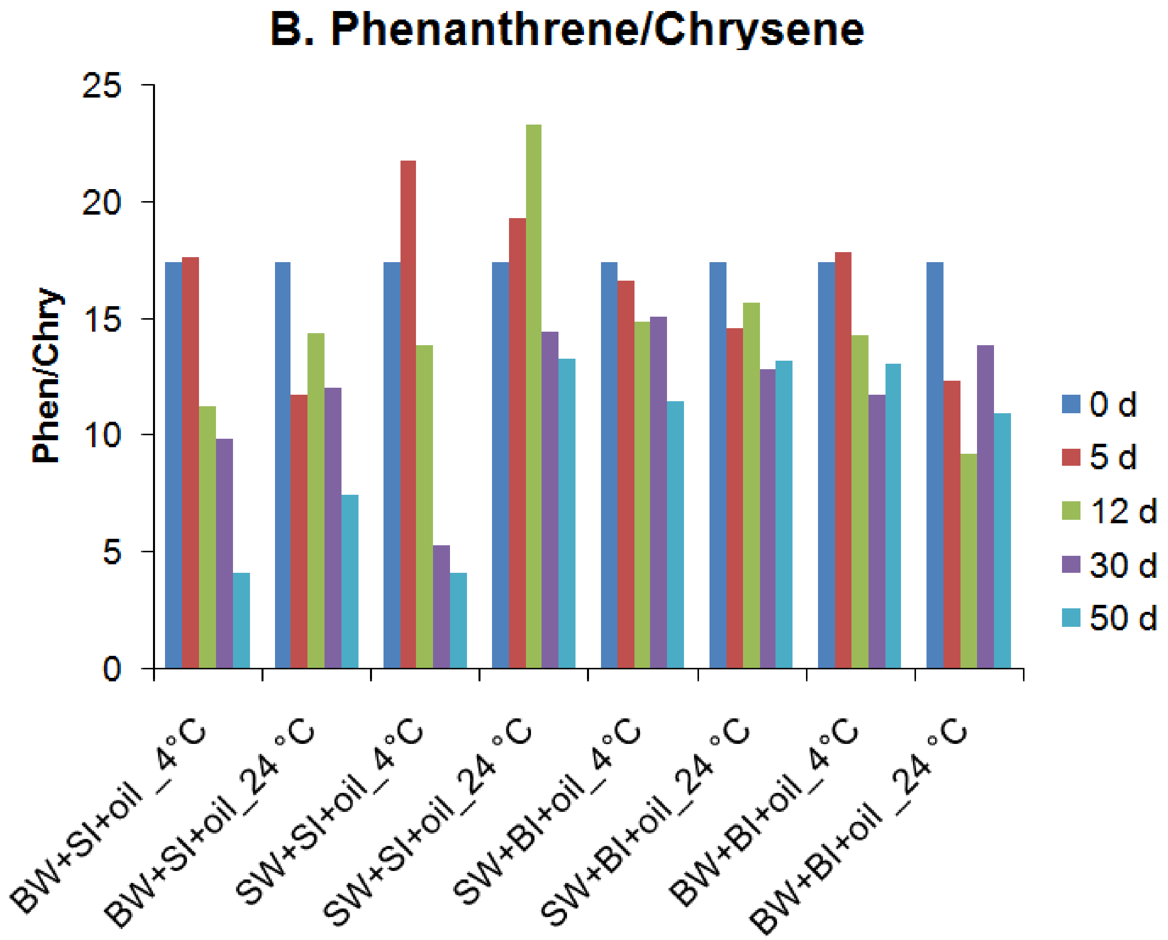

FIGURE S6. ShannonWiener Index(H) of bacterial communities. The indices represent the average of the time series(5,12 and 50 days).

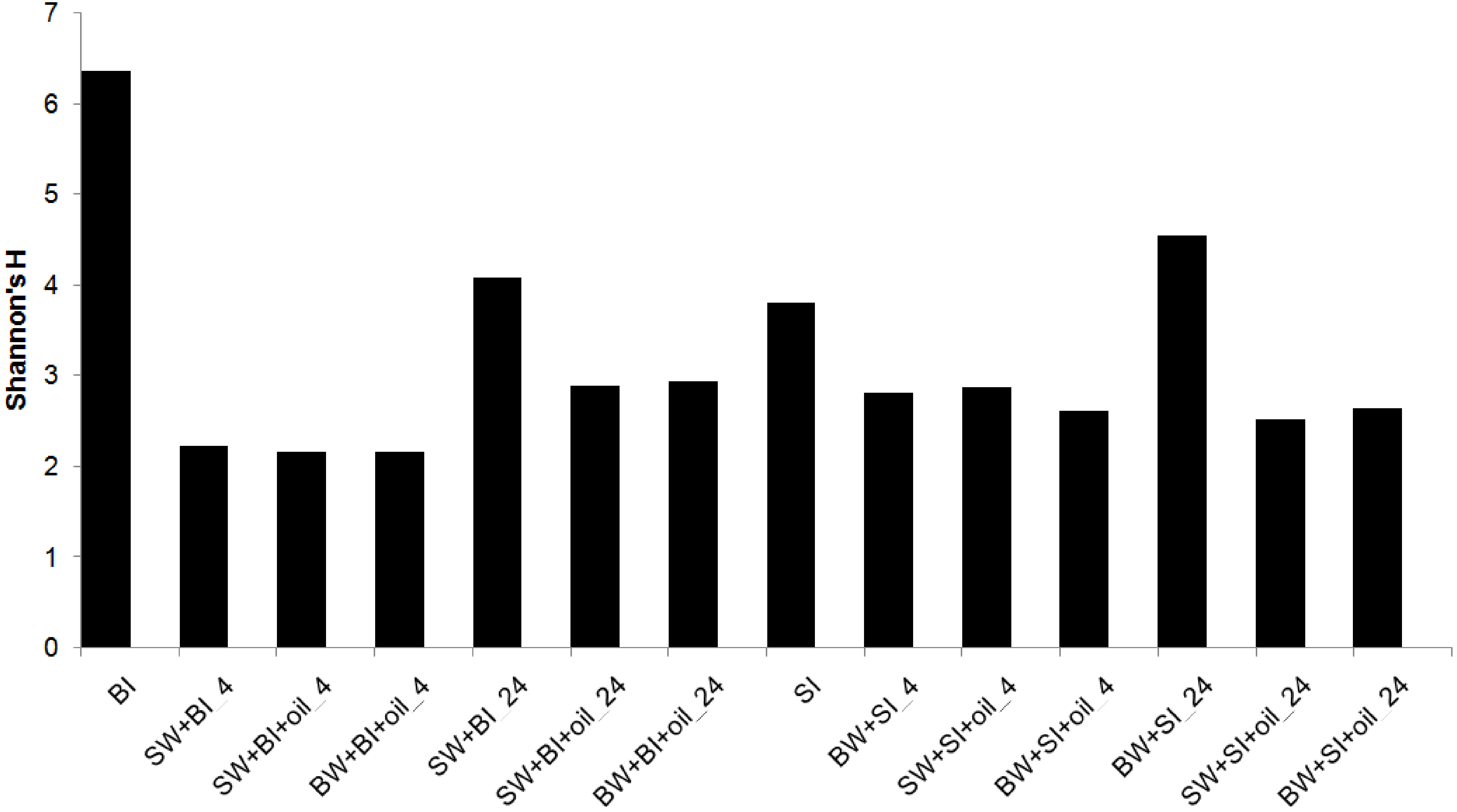

FIGURE S7. Concentrations of (A) nitrate plus nitrite ($NO_3^- + NO_2^-$) (**B**) phosphate (PO_4^{3-}), and (**C**) ammonium (NH_4^+) (**A**), during the incubation experiments. BW: filtered bottom water; BWI: unfiltered bottom water; SW: filtered surface water; SWI: unfiltered surface water.

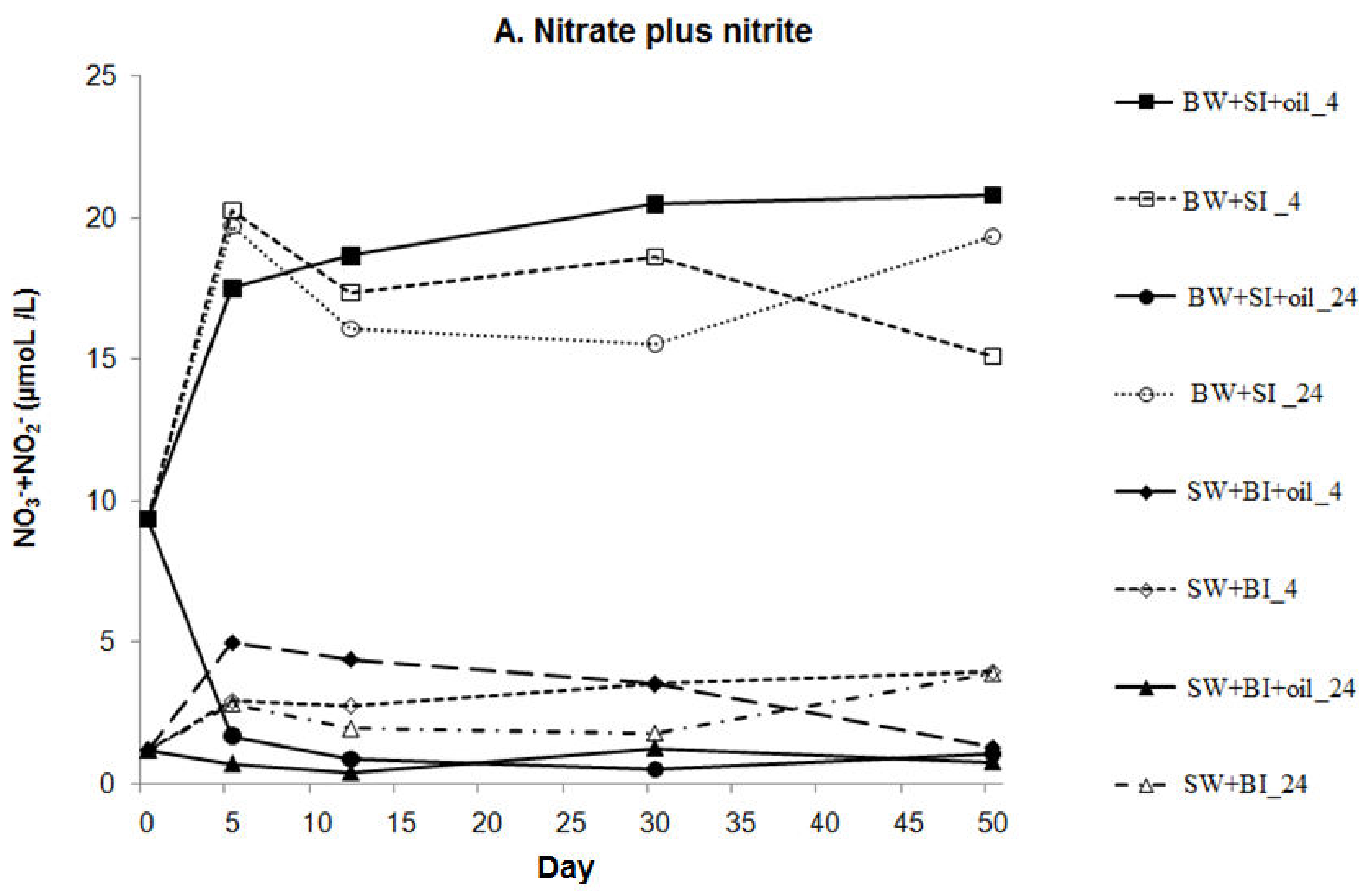

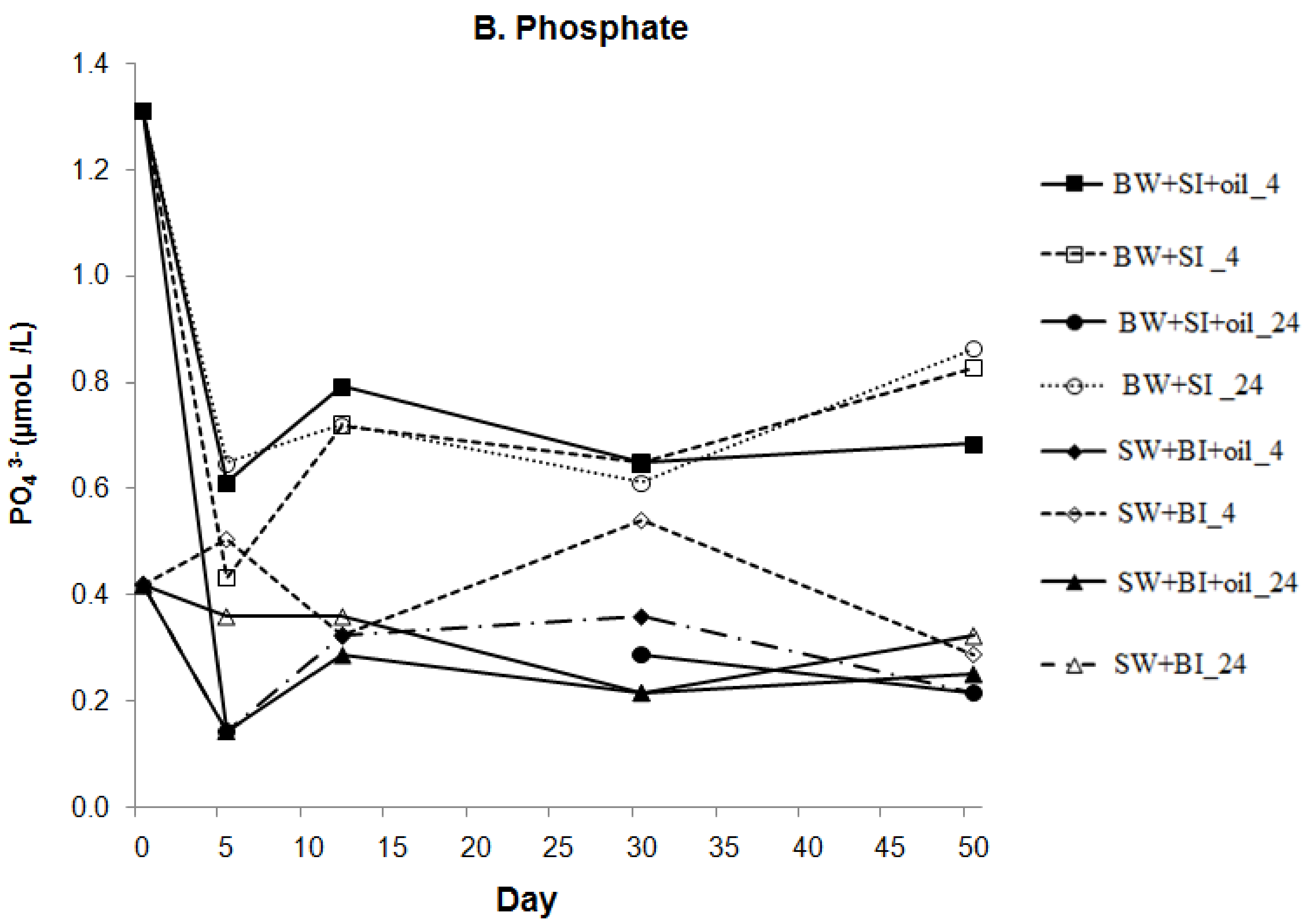
B. Phosphate
1.4
1.2
1.0
0.8
0.6
0.4
0.2
0.0
0
5
10
15
20
25
30
35
40
45
50
Day
PO4 3- (μmoL /L)
BW+SI+oil_4
BW+SI _4
BW+SI+oil_24
BW+SI _24
SW+BI+oil_4
SW+BI_4
SW+BI+oil_24
SW+BI_24

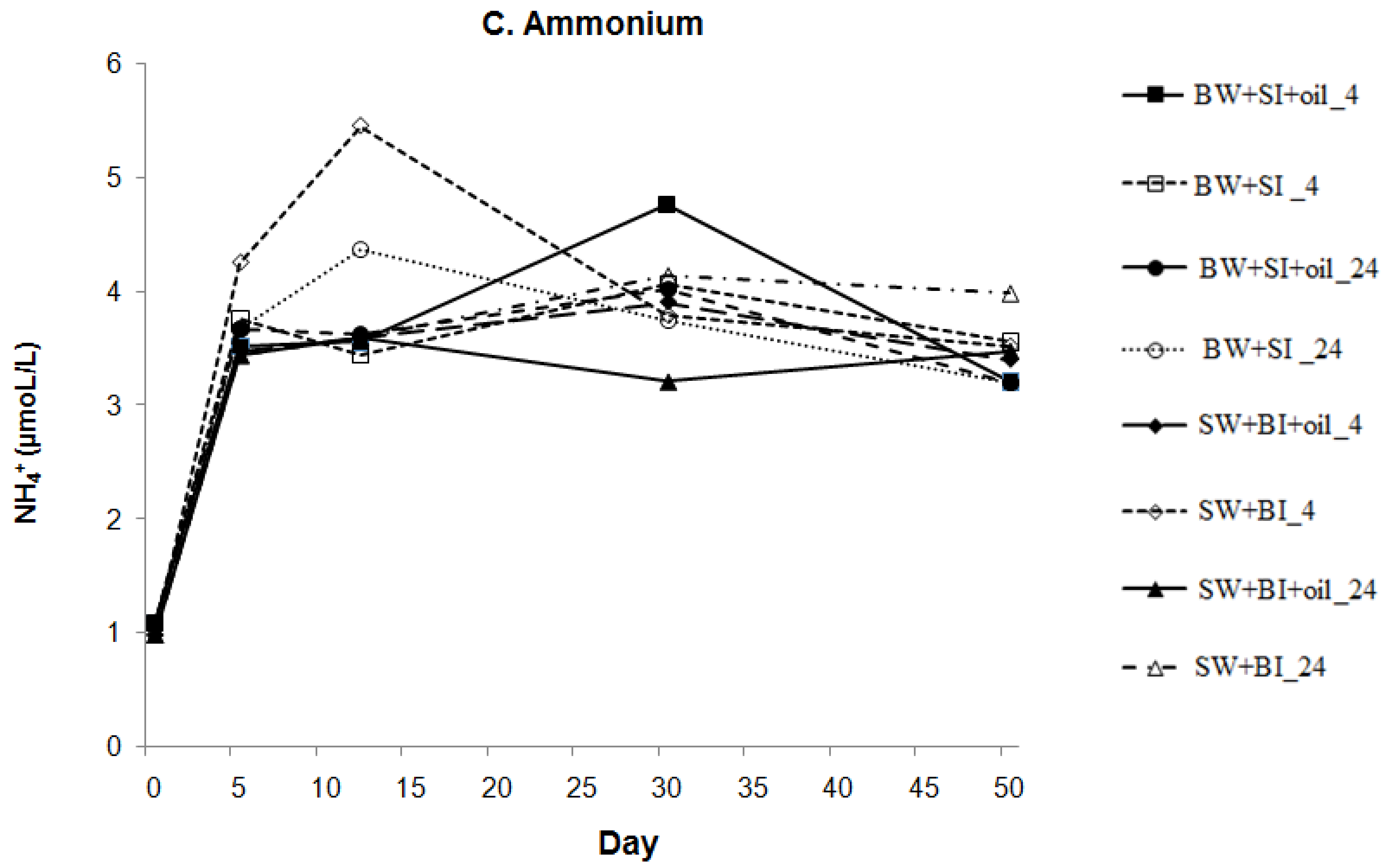
C. Ammonium
NH4+ (μmoL/L)
6
5
4
3
2
1
0
0
5
10
15
20
25
30
35
40
45
50
Day
BW+SI+oil_4
BW+SI _4
BW+SI+oil_24
BW+SI _24
SW+BI+oil_4
SW+BI_4
SW+BI+oil_24
SW+BI_24
